# Rapid detection of neutralizing antibodies to SARS-CoV-2 variants in post-vaccination sera

**DOI:** 10.1093/jmcb/mjab050

**Published:** 2021-08-27

**Authors:** Kei Miyakawa, Sundararaj Stanleyraj Jeremiah, Hideaki Kato, Yutaro Yamaoka, Hirofumi Go, Satoshi Yajima, Tomoko Shimada, Takahiro Mihara, Atsushi Goto, Takeharu Yamanaka, Akihide Ryo

**Affiliations:** Department of Microbiology, Yokohama City University School of Medicine, Yokohama, Japan; Department of Microbiology, Yokohama City University School of Medicine, Yokohama, Japan; Infection Prevention and Control Department, Yokohama City University Hospital, Yokohama, Japan; Department of Microbiology, Yokohama City University School of Medicine, Yokohama, Japan; Life Science Laboratory, Technology and Development Division, Kanto Chemical Co., Inc., Isehara, Japan; Department of Biostatistics, Yokohama City University Graduate School of Medicine, Yokohama, Japan; Clinical Laboratory Department, Yokohama City University Hospital, Yokohama, Japan; Nursing Department, Yokohama City University Hospital, Yokohama, Japan; Department of Health Data Science, Yokohama City University Graduate School of Data Science, Yokohama, Japan; Department of Health Data Science, Yokohama City University Graduate School of Data Science, Yokohama, Japan; Department of Biostatistics, Yokohama City University Graduate School of Medicine, Yokohama, Japan; Department of Health Data Science, Yokohama City University Graduate School of Data Science, Yokohama, Japan; Department of Microbiology, Yokohama City University School of Medicine, Yokohama, Japan

The uncontrolled spread of the coronavirus disease 2019 (COVID-19) pandemic has led to the emergence of different severe acute respiratory syndrome coronavirus 2 (SARS-CoV-2) variants across the globe. The ongoing global vaccination strategy to curtail the COVID-19 juggernaut is threatened by the rapidly spreading variants of concern (VOC) and other regional mutants, which are less responsive to neutralization by infection- or vaccine-derived antibodies (Gomez et al., 2021; Wang et al., 2021).

We have previously developed the HiBiT-based virus-like particle neutralization test (hiVNT) system, which detects SARS-CoV-2 neutralizing antibodies (nAb) in sera in less than 3 h (Miyakawa et al., 2020). It uses lentivirus-based virus-like particles (VLPs) incorporated with the NanoLuc fragment peptide, HiBiT. Viral entry into reporter cells that express LgBiT intracellularly allows the viral HiBiT to fuse with the LgBiT to reconstitute whole NanoLuc luciferase, which is readily detected by luminometer (Schwinn et al., 2018; Miyakawa et al., 2020). The hiVNT assay is capable of high throughput and can be easily carried out in a low-biosafety setting. The quantitative hiVNT assay also correlates well with the gold standard neutralization assay using authentic SARS-CoV-2 (Supplementary Figure S1). In this study, we have modified the hiVNT for rapid qualitative screening of nAb to multiple VOC of SARS-CoV-2. Using qualitative hiVNT, we assess the neutralizing efficacy of the BNT162b2 mRNA vaccine (Pfizer–BioNTech) on a panel of seven epidemiologically relevant SARS-CoV-2 variants.

Using the spikes of the prototype virus (D614G) and seven variants (B.1.1.7, B.1.351, P.1, R.1, B.1.617, B.1.429, and B.1.526) (Figure 1A), we generated HiBiT-containing VLPs (hiVLPs). If the serum nAb neutralize the mutant hiVLPs, their entry into the reporter cells is prevented, reducing the luminescence emitted (Figure 1B). We optimized the conditions of the hiVNT using sera of COVID-19 patients (*n *=* *97) and healthy individuals (*n *=* *43) with pre-defined neutralization titers (NT50) by the pseudovirus-based neutralization assay (Goto et al., 2021; Wang et al., 2021). Specifically, each of these sera was assayed by the HIV-based pseudovirus method to determine the neutralizing titer (pvNT50) and classified as pvNT50 = 50 or less (<50), 51–200, 201–500, 501–1000, and 1000 or more (>1000). The same sera were then assayed by qualitative hiVNT method with hiVLP (D614G prototype) to calculate the percentage inhibition. At a fixed serum dilution of 1 in 20, the hiVNT assay could detect the nAb titers corresponding to pvNT50 of ∼50, which is the positive cutoff in the standard pseudovirus neutralization assay. We then determined that a sample possessed neutralizing activity if it showed >40% luminescence signal inhibition in the hiVNT assay (Figure 1C). The receiver operating characteristic curve showed that this cutoff value of 40% can detect the presence of nAb with a high degree of accuracy (Figure 1D). This modification of performing the hiVNT at a fixed dilution makes the procedure less laborious and reduces the turn-around time, while achieving performance similar to the standard neutralization assays.

**Figure 1:**
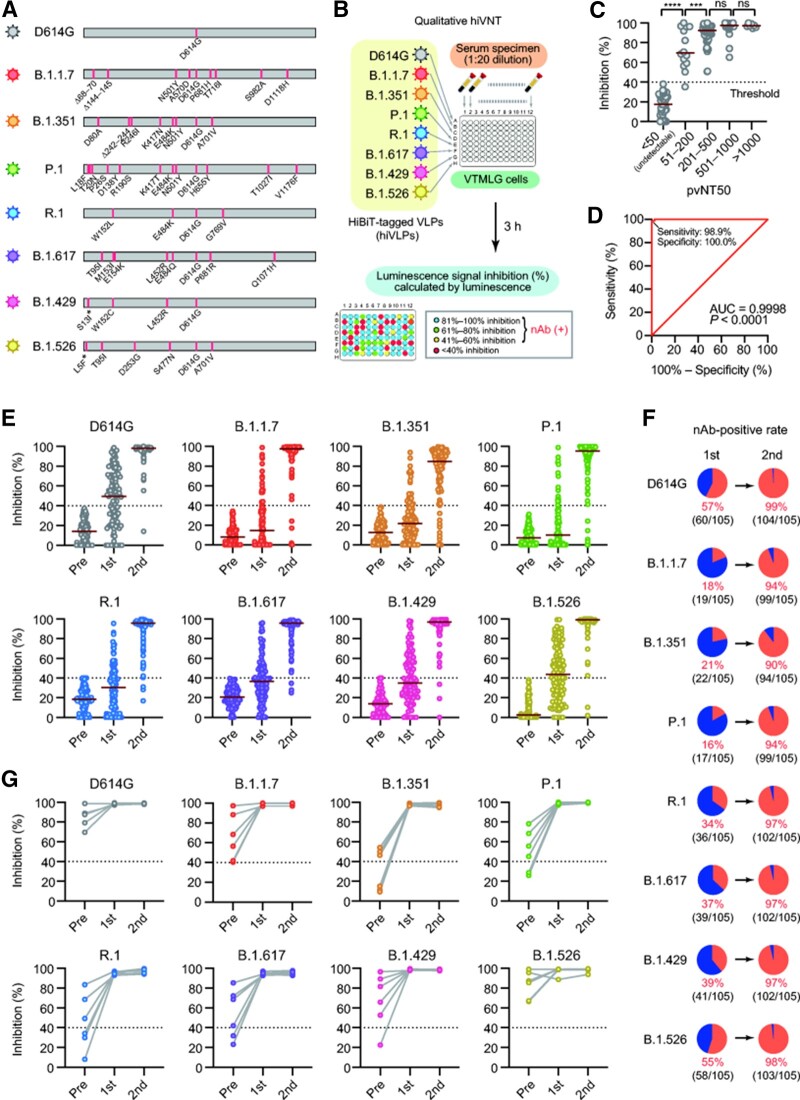
Modified hiVNT for qualitative detection of SARS-CoV-2 nAb. (**A**) Spike mutations of indicated SARS-CoV-2 variants used in this study. Since the N-terminus of the spike has been replaced with the CD5 signal sequence for incorporation into VLPs, the mutations marked with an asterisk are not applied in this study. (**B**) Eight strains of hiVLPs were added to VeroE6/TMPRSS2-LgBiT cells (VTMLG) in the presence of 5% serum. Three hours later, cells were washed and measured to detect the luminescence signal inhibition of each serum. (**C**) Correlation of hiVNT with pseudovirus NT. Each of COVID-19 sera was assayed by the HIV-based pseudovirus NT to determine the neutralizing titer (pvNT50) and classified as shown in x-axis. The same sera were then assayed by qualitative hiVNT method with hiVLP (D614G prototype) to calculate the percentage inhibition as shown in y-axis. Sera with the luminescence signal inhibition <40% can be deemed negative for nAb. ***P <* *0.001, ****P <* *0.0001, ns, not significant, two-tailed unpaired *t*-test. (**D**) Receiver operating characteristic curve of hiVNT. Area under the curve (AUC) is also shown. The cutoff value of 40% detects the presence of nAb with a high degree of accuracy (sensitivity 98.9%; specificity 100%). (**E**–**G**) Positive conversion of nAb to SARS-CoV-2 variants in post-vaccination sera from previously uninfected (**E **and** F**) or post-COVID-19 (**G**). Red horizontal bars represent median of luminescence signal inhibition. The positive rate of nAb against indicated strain is shown in a pie chart (**F**). Pre, first, and second indicate before vaccination, after first does, and after second dose, respectively.

The study samples were collected from otherwise healthy medical personnel who were administered with two doses of the vaccine 3 weeks apart. The mean age of the participants was 42 years (ranging 24–62 years). Serum of the vaccinees was collected at three instances: before the first dose, 2 weeks after the first dose, and 1 week after the second dose. All sera collected prior first vaccine shot were checked for seropositivity using NP-IgG detection assay (Kubo et al., 2020) to get two vaccine recipient groups: previously uninfected (*n *=* *105) and post-COVID-19 (*n *=* *6).

Among the previously uninfected vaccine recipients, 57.1% (60/105) showed a positive conversion for nAb against the conventional strain (D614G) after the first dose. However, the nAb against the mutant strains (except B.1.526) were much lower (16.2%–39.0%) (Figure 1E and F).

A steady increase in nAb was observed after the first dose, followed by the achievement of significant nAb levels against all mutants in most people after the second dose (Figure 1E and F). The vaccine-derived nAb possessed high median inhibition rates of >94% for all variants except B.1.351, which was 89.5% (94/105). All the six individuals with past SARS-CoV-2 infection achieved significant levels of nAb after the first dose despite some possessing nAb below threshold prior initial vaccination (Figure 1G).

Fitter mutants of SARS-CoV-2 of varying dissimilarity to the initial wild-type outbreak strain have emerged and disseminated rapidly. This has raised concerns about the efficacy of the currently available vaccines, which rely on the antigenic determinants of the older virus to provide protective immunity. Among the currently studied mutants, those possessing the E484K mutation such as B.1.351, P.1, and R.1 are notorious for escaping infection- and vaccine-derived nAb (Gomez et al., 2021).

To clear this doubt on vaccine escape, we developed an extensive mutant panel including the recently surging B.1.617 double mutant (E484Q and L452R) (https://outbreak.info/), which is presumed to have higher propensity to escape nAb. In this first report of vaccine efficacy assessment from Japan, we reveal that the BNT162b2 mRNA vaccine has adequate efficacy against the epidemiologically relevant SARS-CoV-2 variants. Vaccine escape was noted in all mutants but only in a minor fraction, which was higher for the B.1.351 mutant. Albeit a small sample size, we observed that one dose of vaccine was sufficient to elicit protective humoral response in previously infected people as documented by previous reports (Stamatatos et al., 2021). Despite the presence of their corresponding nAb in the serum, mutants can cause vaccine breakthrough infections, suggesting that the circulating nAb might not offer protection at mucosal surfaces to prevent infection (Hacisuleyman et al., 2021).

In the current study, we show that the BNT162b2 mRNA vaccine can activate humoral immunity against the major SARS-CoV-2 mutants that are currently in circulation. Moreover, this study shows that people who have recovered from COVID-19 could be effectively protected with a single round of vaccination, corroborating the claim of Frieman et al. (2021). Our modified hiVNT possesses the advantages of being rapid, safe, concise, and comprehensive over the conventional neutralization assays. However, this modified hiVNT assay is qualitative and cannot quantify the nAb titers at relatively higher levels that maybe relevant to their long-term persistence and duration of protection. Using a panel of seven SARS-CoV-2 variants and a single prototype virus, our modified hiVNT would be useful for large-scale community-wide testing to detect protective immunity that may confer vaccine/immune passport in the ongoing COVID-19 pandemic. The selection pressure exerted by the current vaccines would cause the evolution of vaccine-escape mutants, which must be monitored in the future and for which the hiVNT assay developed using the spikes of new variants could come in handy.

*[Supplementary material is available at Journal of Molecular Cell Biology online. We acknowledge all medical staff involved in the study. We thank Kenji Yoshihara, Kazuo Horikawa, and Natsumi Takaira for their technical assistance. This study was supported by a grant‐in‐aid from the Japan Agency for Medical Research and Development (JP19fk0108110, JP20he0522001, and JP21fk0108104) to A.R. Y.Y. is a current employee of Kanto Chemical Co., Inc. K.M. designed and performed the research, analyzed the data, and wrote the manuscript; S.S.J.* *analyzed the data and wrote the manuscript; Y.Y. performed the research and analyzed the data; H.K., H.G., S.Y., T.S., T.M., and A.G. recruited the participants and collected the specimens; H.K., H.G., and T.Y. analyzed the data; A.R. directed the research, analyzed the data, and wrote the manuscript.]*

## Supplementary Material

mjab050_Supplementary_Data
