# Supplementary material for: Rapid detection of neutralizing antibodies to SARS-CoV-2 variants in post-vaccination sera
Source: J Mol Cell Biol. 2021 Aug 27;13(12):918–20. doi: 10.1093/jmcb/mjab050 (PMC8800509; doi:10.1093/jmcb/mjab050)
Supplement: mjab050_Supplementary_Data [file mjab050_supplementary_data.pdf]

Supplementary material

Rapid detection of neutralizing antibodies to SARS-CoV-2 variants in post-vaccination sera

Kei Miyakawa[1], Jeremiah Sundararaj Stanleyraj[1], Hideaki Kato[2], Yutaro Yamaoka[1,3], Hirofumi Go[4], Satoshi Yajima[5], Tomoko Shimada[6], Takahiro Mihara[7], Atsushi Goto[7], Takeharu Yamanaka[4,7], and Akihide Ryo[1,*]

[1]Department of Microbiology, Yokohama City University School of Medicine, Yokohama, Japan

[2]Infection Prevention and Control Department, Yokohama City University Hospital, Yokohama, Japan

[3]Life Science Laboratory, Technology and Development Division, Kanto Chemical Co., Inc., Isehara, Japan

[4]Department of Biostatistics, Yokohama City University Graduate School of Medicine, Yokohama, Japan

[5]Clinical Laboratory Department, Yokohama City University Hospital, Yokohama, Japan

[6]Nursing Department, Yokohama City University Hospital, Yokohama, Japan

[7]Department of Health Data Science, Yokohama City University Graduate School of Data Science, Yokohama, Japan

*Correspondence to: Akihide Ryo, M.D., Ph.D

Department of Microbiology, School of Medicine, Yokohama City University

3-9, Fukuura, Kanazawa, Yokohama, Japan, 236-0004

E-mail: aryo@yokohama-cu.ac.jp

Phone: +81-45-787-2602

Supplementary Figure

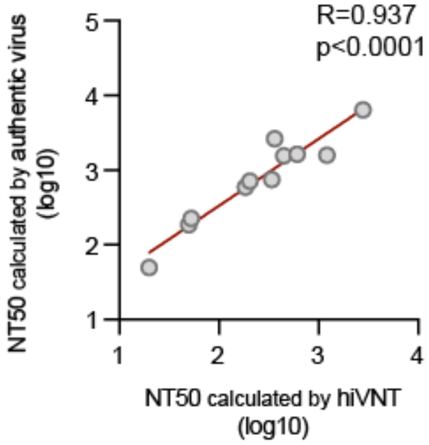

Supplementary Figure 1. Correlation between hiVNT and authentic virus NT.

Scatterplot of neutralizing titers (NT50) calculated from the neutralization analysis using the hiVNT against NT50 calculated from the assay using the authentic SARS-CoV-2 in a validation study.

Materials and Methods

Ethics statement

This study was approved by Yokohama City University Certified Institutional Review Board (Reference No. B160800009, B200600115, B210300001), and the protocols used in the study were approved by the ethics committee. Written informed consent was obtained from all the participants.

Production of hiVLP

HEK293 cells (ATCC #CRL-1573), VeroE6/TMPRSS2 cells (JCRB #1819), and VeroE6/TMPRSS2-LgBiT (VTMLG) cells (Miyakawa et al., 2021) were cultured in DMEM containing 10% FBS. Plasmids encoding HIV-GagPol-HiBiT and SARS-CoV-2 spike were described previously (Miyakawa et al., 2021). Spike mutants were generated by standard mutagenesis procedures. hiVLP was produced by transient transfection of HEK293 cells with pHIV-GagPol-HiBiT and pSARS2-Spike-FLAG at a ratio of 1:1. Culture supernatants containing hiVLPs were collected 48 hours after transfection and filtered through a 0.45 µm Millex-HV filter (Merck).

Modified hiVNT

VeroE6/TMPRSS2-LgBiT cells seeded in 96-well plates were washed and inoculated with hiVLP stocks (50 µl) containing diluted serum (1:20). At 3 hours after inoculation, cells were washed with PBS and treated with 100 µl of PBS containing DrkBiT peptide (1 µM) for 2 minutes. Cells were then added with 25 µl of 1x Nano-Glo Live Cell Substrate (Promega). Luciferase activity is measured with GloMax Discover System (Promega).

The luminescence signal inhibition (%) was calculated as follows:

$$\frac{\text{RLU (without serum)} - \text{RLU (with serum)}}{\text{RLU (without serum)} - \text{RLU (blank)}} \times 100$$

Neutralizing assay using authentic SARS-CoV-2

SARS-CoV-2 (JPN/TY/WK-521) was obtained from National Institute of Infectious Diseases, Japan. For neutralizing assay, VeroE6/TMPRSS2 cells seeded in 96-well plates were washed and infected with 100 µl of medium containing SARS-CoV-2 (moi = 0.05) and five-fold serially diluted serum (1:50 to 1:31250 dilution). At 48 hours after infection, cells

were washed and added with 40 µl of CellTiter-Glo Substrate (Promega). Cell viability is measured with GloMax Discover System (Promega).

Neutralizing assay using HIV-based pseudovirus

Pseudotype lentivirus was produced by transient transfection of HEK293 cells with pNL4-3.Luc.R-E- and pSARS2-Spike-FLAG at a ratio of 1:1. Culture supernatants containing lentiviruses were collected 48 hours after transfection and filtered through a 0.45 µm Millex-HV filter (Merck). For neutralizing assay, VeroE6/TMPRSS2 cells seeded in 96-well plates were washed and inoculated with 100 µl of medium containing lentivirus stocks (20 µl) and five-fold serially diluted serum (1:50 to 1:31250 dilution). At 48 hours after inoculation, cells were washed and added with 40 µl of Bright-Glo Substrate (Promega). Luciferase activity is measured with GloMax Discover System (Promega).

Statistical analysis

The statistical significance of differences between two groups was evaluated by two-tailed unpaired t-test in the Prism 8 software (GraphPad).

References

Miyakawa, K., Jeremiah, S.S., Ohtake, N., et al. (2020). Rapid quantitative screening assay for SARS-CoV-2 neutralizing antibodies using HiBiT-tagged virus-like particles. J. Mol. Cell Biol. *12*, 987-990.
